# Automatic Detection of Calcaneal-Fifth Metatarsal Angle Using Radiograph: A Computer-Aided Diagnosis of Flat Foot for Military New Recruits in Taiwan

**DOI:** 10.1371/journal.pone.0131387

**Published:** 2015-06-30

**Authors:** Chin-Hua Yang, Kuei-Ting Chou, Mu-Bai Chung, K. S. Chuang, Tzung-Chi Huang

**Affiliations:** 1 Department of Radiology, Tao Yuan General Hospital, Ministry of Health and Welfare, Taoyuan City, Taiwan; 2 Department of Biomedical Engineering and Environment Science, National Tsing Hua University, Hsinchu City, Taiwan; 3 Department of Biomedical Imaging and Radiological Science, China Medical University, Taichung City, Taiwan; 4 Department of Biomedical Informatics, Asia University, Taichung City, Taiwan; Bern University of Applied Sciences, SWITZERLAND

## Abstract

Flatfoot (pes planus) is one of the most important physical examination items for military new recruits in Taiwan. Currently, the diagnosis of flatfoot is mainly based on radiographic examination of the calcaneal-fifth metatarsal (CA–MT5) angle, also known as the arch angle. However, manual measurement of the arch angle is time-consuming and often inconsistent between different examiners. In this study, seventy male military new recruits were studied. Lateral radiographic images of their right and left feet were obtained, and mutual information (MI) registration was used to automatically calculate the arch angle. Images of two critical bones, the calcaneus and the fifth metatarsal bone, were isolated from the lateral radiographs to form reference images, and were then compared with template images to calculate the arch angle. The result of this computer-calculated arch angle was compared with manual measurement results from two radiologists, which showed that our automatic arch angle measurement method had a high consistency. In addition, this method had a high accuracy of 97% and 96% as compared with the measurements of radiologists A and B, respectively. The findings indicated that our MI registration measurement method cannot only accurately measure the CA–MT5 angle, but also saves time and reduces human error. This method can increase the consistency of arch angle measurement and has potential clinical application for the diagnosis of flatfoot.

## Introduction

The foot is an anatomically complex structure, consisting of 57 joints, 32 muscles and tendons, and 108 ligaments. These elements are responsible for controlling the movement of the foot. As the medial longitudinal arch has all of the key elements, it has flexed and curved properties that can adapt to uneven surfaces and transfer the power and body weight to the ground during movement. The medial longitudinal arch absorbs the majority of the shock generated from impact during exercise, which can reduce fatigue and avoid injury [1].

It is estimated that 15–30% of people suffer from flatfoot (pes planus) worldwide [2]. The causes of flatfoot include deformity of the normal anatomic relationship between the talus and calcaneus and abnormal ligamentous tension, which cause calcaneal valgus, vertical talus, and tarsal coalition (e.g., between the calcaneus and the navicular bones and the talus and calcaneus bones)[3, 4]. Patients standing on a flat foot usually have a typical appearance, including the foot rolling inwards, the midfoot turning inwards, and the heel bone leaning inwards. Due to the abnormal foot structure, patients with flatfoot often have problems regarding the even distribution of their body weight on the foot during movement, which may result in overloading of the bones or muscles. As the foot points outward, it often leads to excessive pressure on the joints of the knee, and indirectly causes symptoms such as pain in the posterior tibial tendon, an X-shaped curvature of the legs, and difficulty during long distance walking or running [5]. Because a patient with flatfoot is likely to suffer from leg pain during exercise and standing for long periods of time, flatfoot screening has become one of the most important physical examination items for military new recruits in Taiwan [6]. Parameters obtained using the footprint technique have been used to identify individuals with flatfoot [7, 8]. However, the measurement of footprint parameters requires specific instruments and software, as well as manual drawing of the outline of the footprint. The technique is time-consuming and errors are often introduced. Therefore, currently in clinical application, the most accurate method is to use magnetic resonance imaging (MRI) for the diagnosis of flatfoot, as MRI can be used to obtain a complete three-dimensional structural image of the foot [9]. However, MRI has significant disadvantages, including the high cost and the long duration of the scan, and therefore has not been widely used. Currently, lateral x-ray imaging is used for the diagnosis of flatfoot [10], as it has the advantages of being fast, low-cost, and simple to perform. However, due to a large number of individuals needing to be diagnosed every year, lateral x-ray imaging not only requires a large amount of manpower, but also is often unreliable as a result of human error [11].

Owing to the problems related to the above-mentioned methods, this study aimed to develop a method that can efficiently and accurately measure the arch angle. We proposed a method using cross-mutual information that can automatically measure and calculate the arch angle. Our method used the results measured by two experienced radiologists as the standard, then employed a receiver operating characteristic curve (ROC curve) and a Bland–Altman difference plot (B-A plot) to evaluate the accuracy of our method. This automatic computer-calculated method aimed to reduce human error and reduce the amount of manpower required for the diagnosis of flatfoot.

## Material & Methods

### Participants & data acquisition

**Fig 1:**
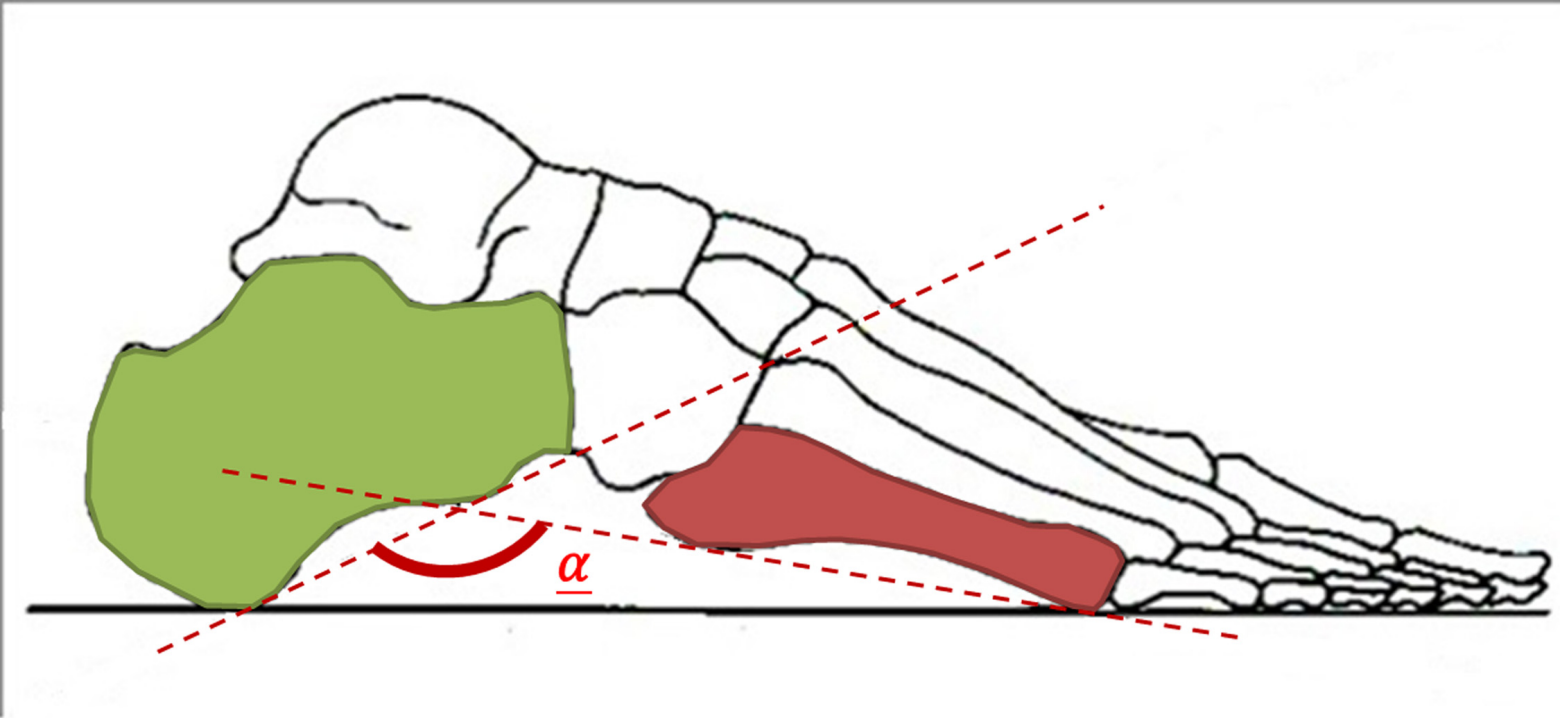
The illustration of calcaneal-fifth metatarsal angle (CA–MT5 angle) α. Diagram of the lateral radiographic images of a participant, which illustrates that the calcaneal-fifth metatarsal angle (CA–MT5 angle) α, also called the arch angle, is defined by lines at the calcaneus and fifth metatarsal. In Taiwan, the criteria for new military conscripts include that individuals are required to serve mandatory service if they have an arch angle <165°, substitute service if they have an angle between 165° to 168°, and are exempt from military service if their arch angle >168°.

This study included 70 Taiwanese military new recruits, with an average age of 21.5 years (range, 19 to 25 years). Lateral radiographic images of both feet were taken using a GE MPC30 X-Ray radiography system (Condition: 100-cm SID, 55 kV and 5 mAs). Measurements of the participants’ calcaneal-fifth metatarsal (CA-MT5) angles (Fig 1) were also obtained by two radiologists (physicians A and B) by analyzing the radiographic images manually, and the results were used for comparison. All patient identifiers were removed from the images for the study. This study was approved by the Institutional Review Board of Tao Yuan General Hospital, Taiwan, and informed consent was waived due to the retrospective nature of the study (TYGH-102053).

### Reference & template images

**Fig 2:**
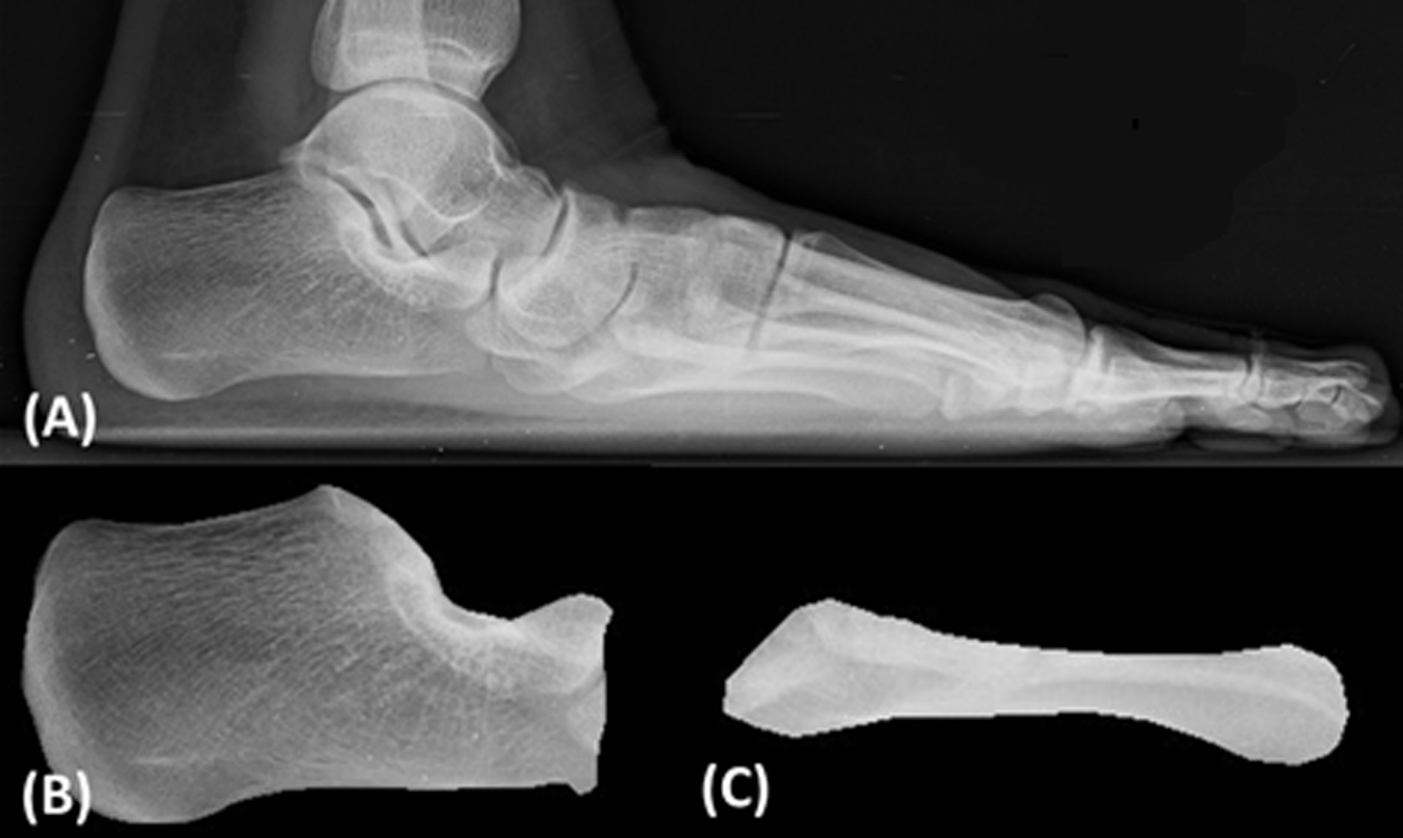
Lateral radiographic image of a participant. (A) The original radiographic image. The reference images of the calcaneus (B) and the fifth metatarsal bone (C) isolated from (A).

**Fig 3:**
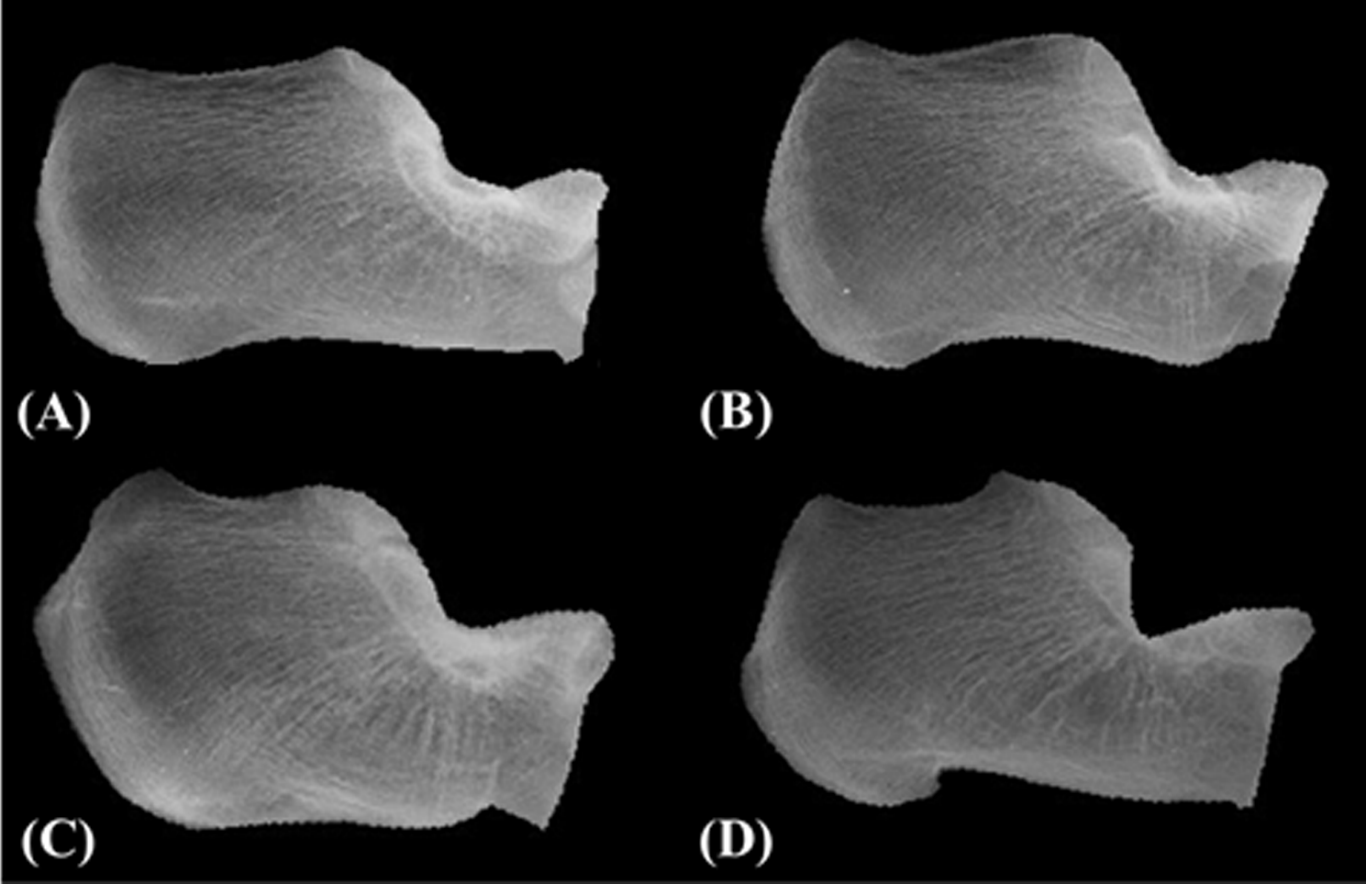
Template images of the calcaneus. The images were classified into four different groups (A, B, C and D).

The automatic CA–MT5 angle measurement method proposed in this study used mutual information (MI) to register the images in order to calculate the CA–MT5 angles of the participants. The radiographic images of two critical bones, the calcaneus and the fifth metatarsal bone, were used as the basis of the analysis. Before automatic measurement, the reference and template images were manually isolated. The images directly isolated from the radiographic images (Fig 2A) were used as the reference images of the calcaneus (Fig 2B) and the fifth metatarsal bone (Fig 2C). For the template images, the images of the calcaneus of all the participants were classified into four different groups. Then, the calcaneal template image was created by using a representative image from each group and rotating until its calcaneal inclination was horizontal (Fig 3), and the template image of the fifth metatarsal was generated by rotating a representative image of the participants to be horizontal.

### Mutual information

Mutual information (MI) was originally used to explain the information transmission for a communication channel. The transmission depends on the input probability distribution. It uses the the input–output relations with a probability matrix, which finds the particular input distribution that maximizes the MI.

The MI between two random variables, *U* and *V*, is denoted *I*(*U*,*V*) to simplify the uncertainity of *U*. On the other hand, *I*(*U*,*V*) represents a formula that contains at least as much information about variable *U* (or *V*) as variable *V* (or *U*). Then, the joint entropy of U and V can be defined as per Eq (1):

$$\text{I}\left(U,V\right)=\sum_{u,v}\;p_{UV}\left(u,v\right)\;\text{log}\frac{p_{UV}\left(u,v\right)}{p_{U}\left(u\right)\cdot p_{V}\left(v\right)}$$ (1),

where *u* and *v* are two random variables, and their entropies, *p*(*u*) and *p*(*v*), are the marginal probability distribution functions, and *p*(*u*, *v*) is the joint probability distribution. Thus, *I*(*U*, *V*) is determined on *p*_*UV*_(*u*, *v*) (the joint probability distribution of *U* and *V*) and *p*_*U*_(*u*)⋅*p*_*v*_(*v*), when U and V are independent.

**Fig 4:**
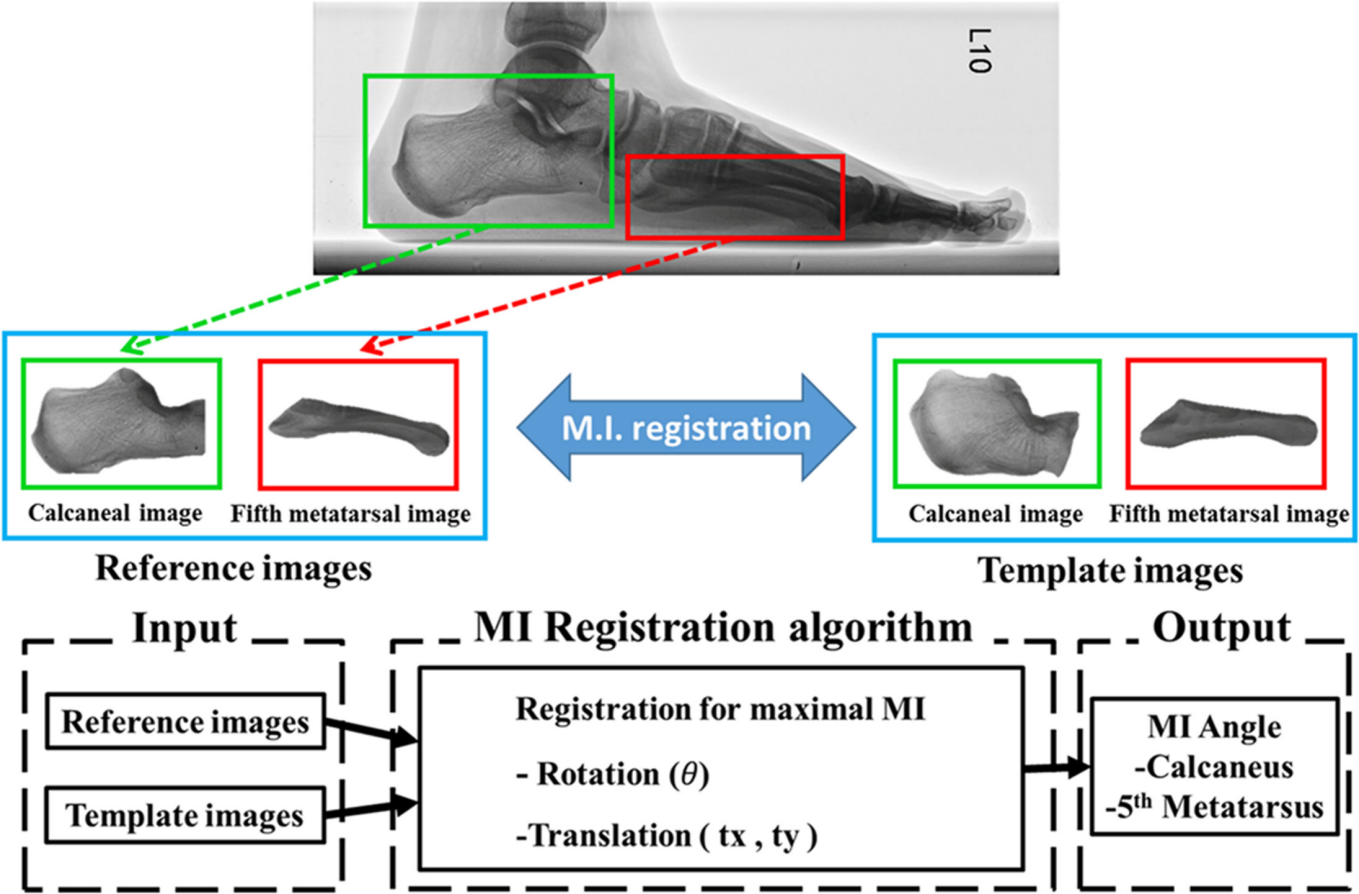
Flowchart of the methodology of the mutual information (MI) registration algorithm. The images were converted into two-dimensional image parameters (*θ*, *tx*, *ty*). Image registration was achieved after repeated calculation until the MI between the template and the reference images was maximized.

In order to measure the CA–MT5 angle of the different subjects to validate whether one has flatfoot, an MI algorithm was used for image registration to maximize the MI values of the reference and template images. A greater MI value indicated that the geometry of the two images was similar. The two-dimensional image was first converted by replacing with rotation angle *θ* and translations in the x and y directions, *tx* and *ty*, as indicated in the following Eq (2):

$$T=\left[\begin{array}{c} x\\ y \end{array}\right]\;;\;T\prime =\left[\begin{array}{c} x+tx\\ y+ty \end{array}\right]=\left[\begin{array}{c} x\prime \\ y\prime \end{array}\right]$$ (2)

$$T\prime \prime =T\prime \;\cdot \;R\left(\theta \right)=\left[\begin{array}{c} x\prime \\ y\prime \end{array}\right]\;\cdot \;\left[\begin{array}{cc} cos\;\theta & -sin\;\theta \\ sin\;\theta & cos\;\theta \end{array}\right]$$,

where *T* is a point on the template image *T*, *T*′ is the position of the point with translations of *tx* and *ty*, and *T*″, the position after rotation at angle *θ*, can be calculated from *T*′ and the rotation matrix, *R*(*θ*). Using this algorithm, rotation and translation of the entire template image can be performed repeatedly, and compared with the reference image. When the maximum MI is obtained, the geometries of the template and reference images are most similar, which is the goal of image registration. When the template image is rotated until the MI between it and the reference image is maximized, the rotation angle represents the calcaneal inclination or the fifth metatarsal inclination to the horizontal line. The angles can then be used to calculate the calcaneal-fifth metatarsal angle (CA-MT5 angle). The flowchart of the methodology of the mutual information (MI) registration algorithm is shown in Fig 4.

### Statistical analysis

The results obtained using the MI registration algorithm were compared with the manual measurement results of two radiologists (physicians A and B). The data were analyzed using SPSS software (SPSS for Windows, release 8.0.0, SPSS Inc, Chicago, USA). In order to validate the accuracy and application of the MI algorithm, a Bland-Altman plot and a receiver operating characteristic curve were used to compare the consistency between the two methods.

## Results

**Fig 5:**
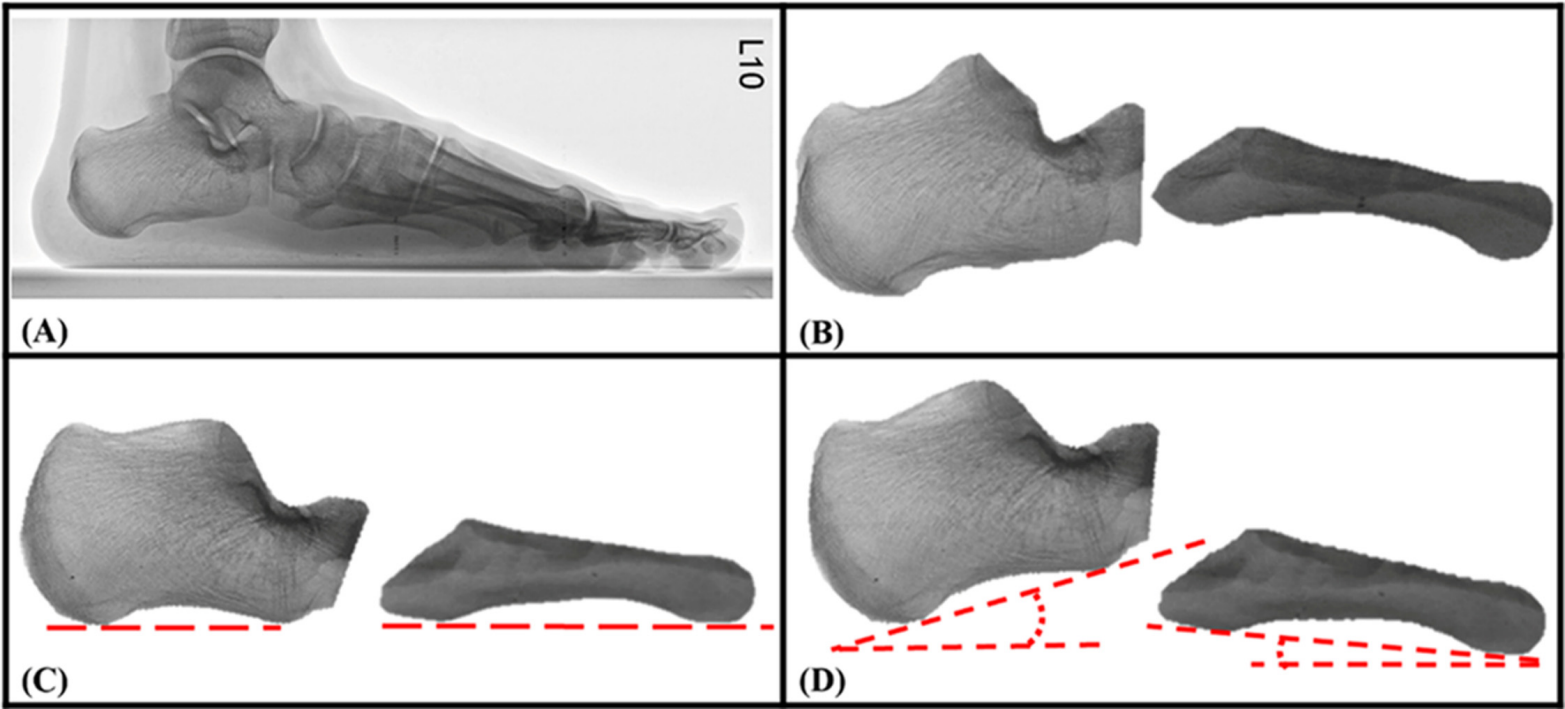
Process of CA–MT5 angle measurement for subject 31 using the MI registration algorithm. (A) Lateral radiographic image of the subject. (B) Reference images of the two critical bones were manually isolated. (C) Generation of the template images (lower edges to be horizontal) and classification (classified as Group C in this case). (D) MI image registration to obtain the angles of inclination of these two bones.

Using the lateral radiographic images of subject 31 as an example (as shown in Fig 5A), images of the critical bones, the calcaneus and the fifth metatarsal bone (Fig 5B), were isolated as the reference images. The reference images of the calcaneus were then rotated to be template images, and they were classified later as Group C template images (Fig 5C). Using the MI algorithm, the template images were moved and rotated until the mutual information between it and the reference image was maximized, which revealed the angle of the straight line at the lower edge of the calcaneus or the fifth metatarsal bone. The CA–MT5 angle was then calculated from the angles of inclination of these two bones. The CA–MT5 angle of subject 31 was 162.0° as calculated by the MI registration algorithm; the manual measurements from radiologists A and B were both 162.6°.

A comparison of the results between the MI registration algorithm and the radiologists is shown in Table 1. The medians of the CA–MT5 angle measurements from radiologists A and B were 163.2° (range, 151.4° ~ 179.0°) and 162.7° (range, 151.1° ~ 176.9°), respectively. On the other hand, the median of the measurements obtained using MI was 164.0° (range, 156.0° ~ 176.0°). The total numbers of participants diagnosed with flatfoot by radiologist A, radiologist B, and using the MI method were 17, 12, and 16, respectively.

**Table 1:** Comparison of the CA-MT5 angle measurements obtained by radiologists A and B and using the MI registration algorithm.

		Dr. A	Dr. B	MI
**CA-MT5 angle**	**Range**	151.4° ~ 179.0°	151.1° ~ 176.9°	156.0° ~ 176.0°
	**Median**	163.2°	162.7°	164.0°
**Flatfoot**		17	12	16
**Non-flatfoot**		53	58	54

Table 2 shows a comparison of the measurement differences among radiologists A and B and the MI registration algorithm. The results demonstrated that the difference was 1° ± 0.96° (Kappa value = 0.023) between radiologists A and B; 2.45° ± 1.79° between radiologist A and MI; and 2.87° ± 2.19° between radiologist B and MI. When the CA-MT5 angle measurement obtained by radiologist A was used as the standard, the accuracy of the measurements obtained by radiologist B and MI was 90% and 96%, respectively.

**Table 2:** Comparison of the difference in the CA-MT5 angle among radiologists A, B, and the MI registration algorithm.

	Dr. A vs. Dr. B	Dr. A vs. MI	Dr. B vs. MI
**CA-MT5 angle mean difference**	1° ± 0.96°	2.45° ± 1.79°	2.87° ± 2.19°
**Accuracy**	94%	97%	96%

**Fig 6:**
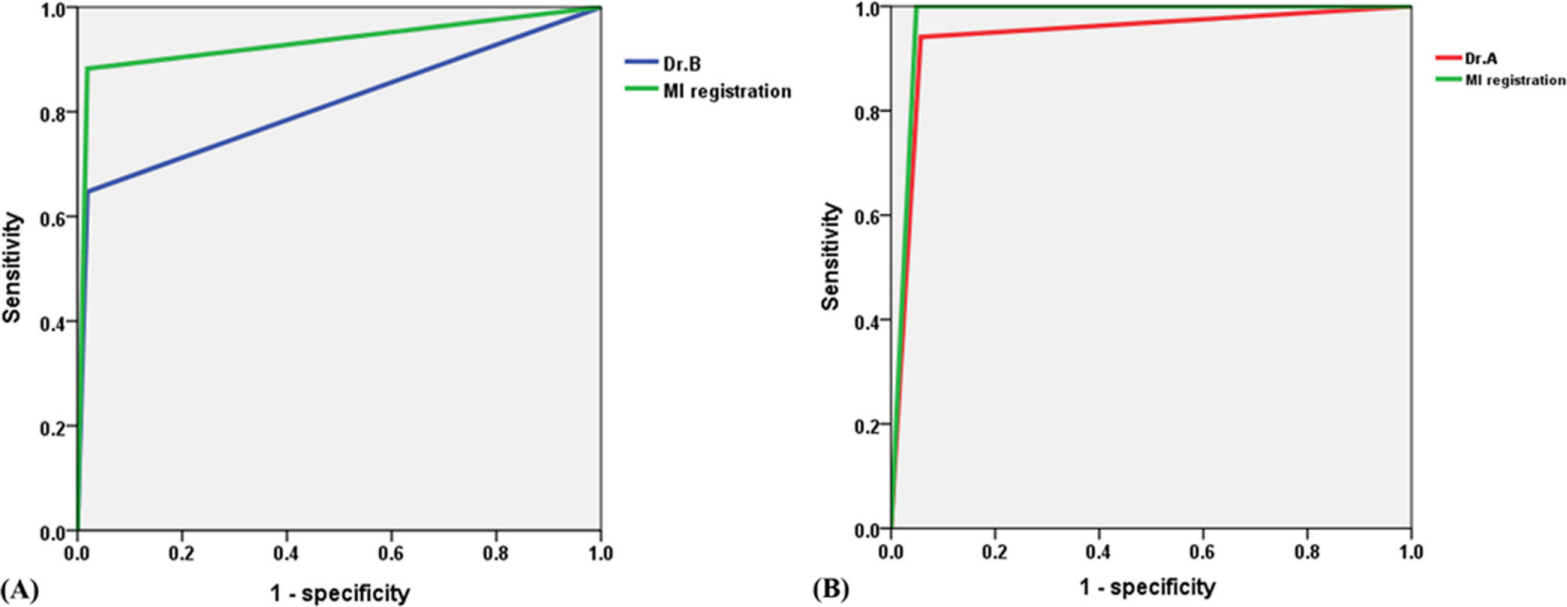
Receiver operating characteristic (ROC) curves of different measurements. (A) ROC curves of radiologist B and MI registration when the measurement of radiologist A was used as the standard. (B) ROC curves of radiologist A and MI registration when the measurement of radiologist B was used as the standard.

Fig 6 shows the receiver operating characteristic (ROC) curve used to analyze the differences between the measurement obtained by MI registration and those of radiologist A or B when the results measured by radiologists A or B were used as the reference. In Fig 6A, when the measurement of radiologist A was used as the standard, the ROC curve showed that the area under the curve of the ROC (AUC ROC) of radiologist B was 81.4%, with a sensitivity of 64.7% and a specificity of 98.1%; and the AUC ROC of MI registration was 93.2%, with a sensitivity of 88.2% and a specificity of 98.1%. When the measurement of radiologist B was used as the standard, the AUC ROC of radiologist A was 94.2%, with a sensitivity of 94.1% and a specificity of 94.3%; and the AUC ROC of MI registration was 97.6%, with a sensitivity of 100% and a specificity of 95.1%.

## Discussion

Clinical measurement of the arch angle from radiographic images using a manual method is prone to error and can result in different diagnoses. As shown in this study, the measurements of radiologists A and B demonstrated a low Kappa value (*κ* = 0.023 <0.2), indicating that the similarity of the results from radiologists A and B was very low. In addition, in a comparison of the accuracy among the three measurements (Table 2), the accuracies of MI registration versus radiologists A or B were both higher than that of radiologist A versus radiologist B. Unlike measurement using the manual method, the present study empolyed a computer-assisted diagnostic approach to perform image registration using MI. After first manually isolating reference images and creating template images rotated to be horizontal, MI registration was used to obtain the angles of inclinations of the two critical bones by a continuous rotation and displacement process. The CA–MT5 angle was then accurately calculated from the angles obtained using this computer-assisted diagnostic method.

**Fig 7:**
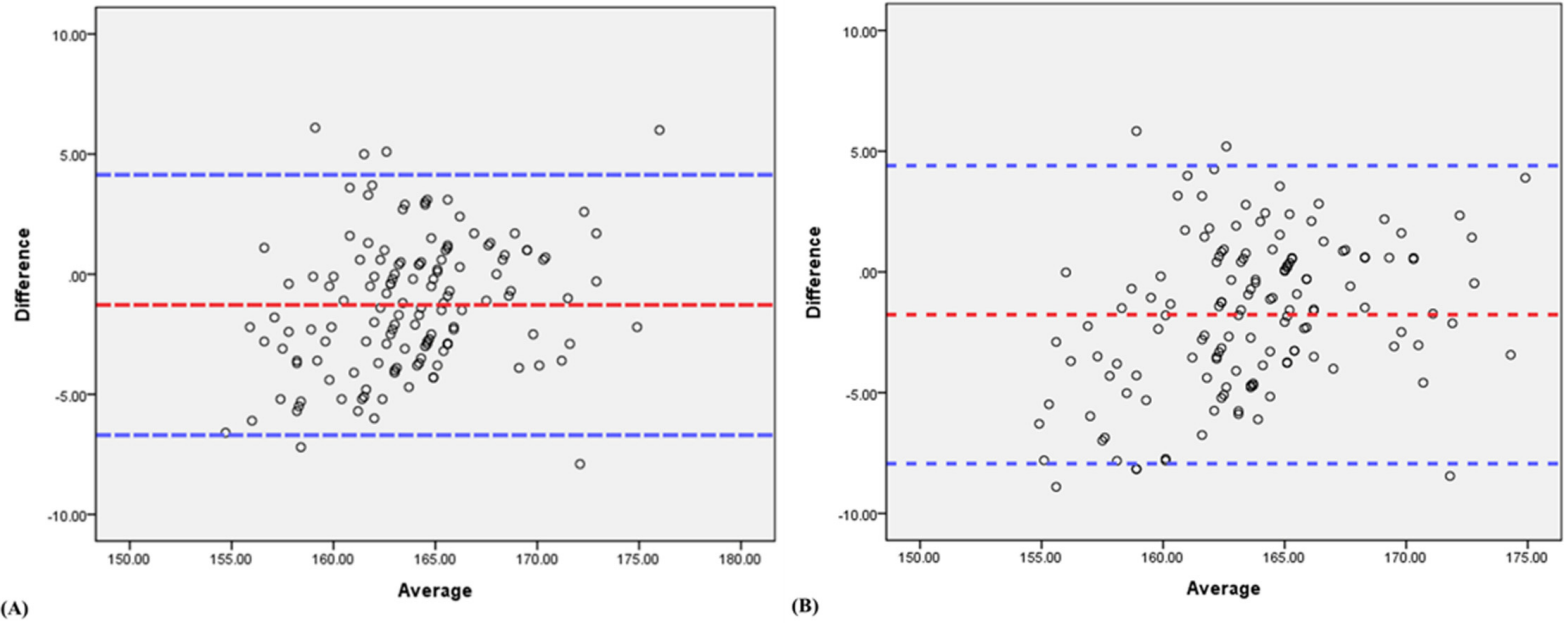
Bland–Altman plot (B-A plot) used to assess the agreement between two measurement methods. (A) Radiologist A vs. MI. (B) Radiologist B vs. MI. The X-axis is the average of the measurements, and the y-axis is the difference between the two methods.

Using a Bland–Altman plot (B-A plot) to analyze the agreement between two different measurement techniques, it was found that the mean and the 95% limits of MI registration compared to the results of radiologists A and B were -1.27, 4.14 ~ -6.70 (MI vs Dr. A, Fig 7A) and -1.77, 4.40 ~ -7.94 (MI vs Dr. B, Fig 7B), respectively. The results indicated differences within the mean ± 1.96 SD, and therefore the MI registration method was not clinically different from the results of either radiologist.

Comparison of the ROC AUC (Fig 6), no matter whether the measurement of radiologist A or radiologist B was used as the reference, showed that MI registration is a better method by which to measure the CA–MT5 angle. In addition, the measurement obtained by MI registration had a higher sensitivity and specificity than those obtained by radiologists A and B.

A recent study by Chang et al. [12] demonstrated the use of a three-dimensional laser scanner in combination with manual measurement to estimate the foot arch. Although our MI registration method was only based on two-dimensional images, the three-dimensional approach requires additional manual anthropometric measurements that require more manpower and are time-consuming to obtain. Human error is also common if the examiners taking the measurements have not been well-trained. The MI registration method proposed in this study has several advantages, including a shorter measurement time, a more simple process, and an increased consistency of diagnosis.

In the past, several methods have been used to estimate the foot arch by footprint methods. These include the arch index [13], footprint index, and arch-length index [14]. Although these footprint methods are cost-effective and time-saving for clinical evaluation, the methods cannot assess the real structure of the foot. Cobey and Sella [11] found that the heights of the arch measured from footprints and radiographic methods are often different. Hawes et al. [14] investigated the relationships between the directly-measured arch height and many footprint parameters, and found that the parameters are invalid as a basis for the prediction of arch height. Saltzman et al. [10] compared anthropometric, footprint and radiographic parameters, and found that radiographic indices are correlated most closely with the acurracy of arch-height measurement. The method proposed in the present study employs two-dimensional radiographic images as the basis and classifies the calcaneus into different groups to improve computer-assisted measurement, which can effectively reduce the false positive rate due to obesity [15, 16].

MI registration is based on image registration to obtain the angles of inclination of the calcaneus and the fifth metatarsal bone. Therefore, image preprocessing to isolate the two critical bones and classification of the calcaneus are important, and are still performed by humans. One might concern that manual processing part would require some labor time and may also introduce errors. However, these two shortcomings can be limited in this study. Identification of bone manually from lateral x-ray imaging is uncomplicated and not the time time-consuming because of the good contrast ratio in the radiographic images. In addition, the bones identification were done by two 10-years-experienced technologists who are familiar with anatomy, which not tend to mistakes. Even if the errors were caused in the manual processing part, the differences in local section of the image might be imperceptive for MI estimation, which was based on global information of the whole image. The classfied template images can then be used in MI registration to accurately measure the arch angle of the subjects, which provides reliable results for clinical diagnosis.

## Conclusion

We have developed a method that uses lateral radiographic images and employs computer-assisted MI registration to accurately calculate the CA-MT5 angle. This method not only reduces the need for manual measurement, which saves time and manpower, but also significantly increases the accuracy, as it eliminates human error. The results of the measurement are of a high consistency, and the method also has a higher sensitivity and specificity than conventional manual measurement by a trained radiologist. Therefore, this automatic calculation method can reduce the manpower requirement in the clinical setting and increase the consistency of measurement, which can provide significant assistance for physicians in the diagnosis of flatfoot. This MI registration method has a high potential for clinical application for flatfoot diagnosis.
